# The Mobile Health App Trustworthiness Checklist: Usability Assessment

**DOI:** 10.2196/16844

**Published:** 2020-07-21

**Authors:** Afua van Haasteren, Effy Vayena, John Powell

**Affiliations:** 1 Health Ethics and Policy Lab - Department of Health Sciences and Technology Swiss Federal Institute of Technology Zurich Switzerland; 2 Nuffield Department of Primary Care Health Sciences University of Oxford Oxford United Kingdom

**Keywords:** checklist, trustworthiness, trust, mobile health apps, validation, survey

## Abstract

**Background:**

The mobile health (mHealth) app trustworthiness (mHAT) checklist was created to identify end users’ opinions on the characteristics of trustworthy mHealth apps and to communicate this information to app developers. To ensure that the checklist is suited for all relevant stakeholders, it is necessary to validate its contents.

**Objective:**

The purpose of this study was to assess the feasibility of the mHAT checklist by modifying its contents according to ratings and suggestions from stakeholders familiar with the process of developing, managing, or curating mHealth apps.

**Methods:**

A 44-item online survey was administered to relevant stakeholders. The survey was largely comprised of the mHAT checklist items, which respondents rated on a 5-point Likert scale, ranging from *completely disagree* (1) to *completely agree* (5).

**Results:**

In total, seven professional backgrounds were represented in the survey: administrators (n=6), health professionals (n=7), information technology personnel (n=6), managers (n=2), marketing personnel (n=3), researchers (n=5), and user experience researchers (n=8). Aside from one checklist item—“the app can inform end users about errors in measurements”—the combined positive ratings (ie, *completely agree* and *agree*) of the checklist items overwhelmingly exceeded the combined negative ratings (ie, *completely disagree* and *disagree*). Meanwhile, two additional items were included in the checklist: (1) business or funding model of the app and (2) details on app uninstallation statistics.

**Conclusions:**

Our results indicate that the mHAT checklist is a valuable resource for a broad range of stakeholders to develop trustworthy mHealth apps. Future studies should examine if the checklist works best for certain mHealth apps or in specific settings.

## Introduction

From self-diagnosis to wellness, mobile health (mHealth) apps have evolved as a conduit for individuals to play more pronounced roles in their own health care [1,2]. Sustaining the uptake of mHealth apps is particularly necessary to realize their potential in health systems. Among others, mHealth apps are perceived as a vehicle to enhance patient-provider communication, boost patient attempts to self-monitor health conditions, as well as to advance patient empowerment [3]. Despite these positive outcomes, several studies have found significant flaws in the privacy, security, and safety claims of several mHealth apps on the market [4,5].

There is evidence to suggest that end users abandon or reject mHealth apps that they perceive as untrustworthy [6-8]. So far, regulatory bodies such as the US Food and Drug Administration have been more concerned about the safety of mHealth apps that purport to be medical devices or their accompanying add-ons [9]. The wellness app space lacks quality assurance frameworks and clear guidance from respective authorities [10]. In light of the relaxed regulatory approach to mHealth apps, evaluation tools have been proposed to assist end users in determining which apps are safe or secure and, thus, which can be trusted [11,12]. Very few of these tools target app developers, although they are vital stakeholders in ensuring that mHealth apps are trustworthy from the outset [13]. To fill this gap, we created the mHealth app trustworthiness (mHAT) checklist via a focus group study with end users of health apps. The mHAT checklist is displayed in Multimedia Appendix 1 and details of the study can be found elsewhere [14].

The purpose of this study was to validate the mHAT checklist by modifying its contents according to ratings and suggestions from stakeholders familiar with the process of developing, managing, or curating mHealth apps to ensure that its contents are applicable. Since the checklist goes beyond the technical aspects of app development, procuring the suggestions of different stakeholders will ensure that it is suited for anyone likely to be involved in app development. The value of validating the checklist was reinforced by a preliminary feedback exercise during the development of the mHAT checklist. Among the six experts with information technology (IT) backgrounds that provided feedback, there was a consensus that the checklist must be validated among a wider range of stakeholders.

## Methods

### Study Design

We designed a cross-sectional online survey to validate the mHAT checklist. Ethical approval for this study was granted by the ETH Zurich (Swiss Federal Institute of Technology) Ethics Commission (EK 2019-N-20). To ensure that this study poses little to no risks to the respondents, we did not require any personal details [15]. Taking part in the survey implied consent. The survey was administered through the SurveyMonkey online survey tool (SurveyMonkey Inc) between March 14 and July 10, 2019.

### Survey Development

The first question in the 44-item open survey screened for eligible respondents to ensure that only individuals with experience in developing apps advanced on to the next page. Another question requested participants’ areas of expertise and was accompanied by four professions—administrators; marketing personnel; software designers, programmers, or developers; and user experience (UX) researchers—as well as a field for free-text answers. To provide an overview of the types of apps created by participants, the next question asked for the function of an app respondents have created within the last 5 years. The remaining 41 survey items consisted of the mHAT checklist items and required answers on a 5-point Likert scale, ranging from *completely disagree* (1) to *completely agree* (5) [16].

Aside from the screening question, which blocked noneligible participants from proceeding further, no other special functions, such as skip logic, were applied to the survey. The number of questions on each page of the survey differed according to the items in each segment of the checklist. Consequently, page 1 contained the 11 questions from the informational content section, whereas page 2 was comprised of the seven questions under *organizational attributes*. SurveyMonkey estimated 10 minutes as the average completion time.

### Participant Recruitment

Our target population was a convenience sample of adults over the age of 18 years who are knowledgeable about the processes involved in developing mobile apps. Among others, administrators (ie, business and systems); marketing personnel; software designers, programmers, and developers; and UX researchers from all geographical locations were eligible for this study. In line with the principles for calculating sample sizes for surveys, the minimum desired sample size was 30 [17]. Participants were recruited by propagating a link of the survey hosted on the SurveyMonkey platform (1) to relevant individuals via email and (2) on social media (ie, Twitter). In line with minimizing access to participants’ personal information, participants’ Internet Protocol (IP) addresses were not tracked, and their computers were also not assigned unique identification numbers.

### Data Analysis

In accordance with recommended practice, the data from the Likert scales were analyzed with descriptive statistics, such as ranks, modes, frequencies, and ranges [16,18]. The frequencies and percentages of the survey items were presented as *positive*, *neutral*, and *negative* using Microsoft Excel. Positive ratings refer to the sum of *completely agree* and *agree*, whereas negative ratings are the sum of *completely disagree* and *disagree*. Meanwhile, the neutral values present the raw ratings of each item on the Likert scale. Only the checklist items with positive ratings (ie, *completely agree* and *agree*) exceeding negative ratings (ie, *completely disagree* and *disagree*) were retained. The data derived from the open-ended questions were analyzed by content analysis in NVivo 12 (QSR International) [19]. Novel suggestions of items to include in the checklist were catalogued accordingly.

## Results

### Participant Characteristics

Of the 144 individuals that responded to calls to participate in this survey, 49 (34.0%) with some professional experience in developing mobile apps were eligible to participate. Out of these 49 participants, 23 (47%) completed the survey in its entirety, while 26 (53%) others completed it partially.

**Figure 1:**
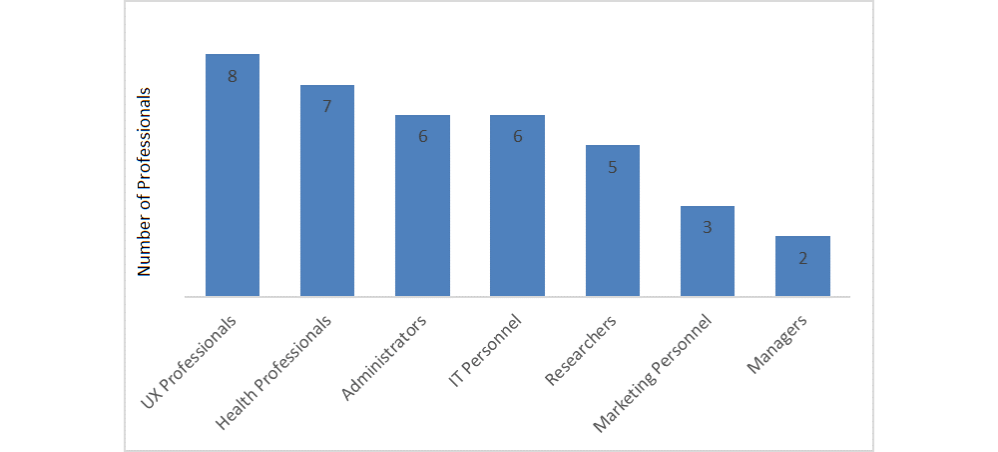
Professional expertise of respondents (n=37). IT: information technology; UX: user experience.

Overall, 22 respondents indicated their professional expertise among the four fixed-choice professions provided in the survey: 6 (27%) administrators; 3 (14%) marketing personnel; 5 (23%) software designers, programmers, or developers; and 8 (36%) UX researchers. Another 15 free-text responses showed additional professional backgrounds: 7 (47%) health professionals, 5 (33%) researchers, 2 (13%) managers, and 1 (7%) IT professional. Figure 1 presents the frequencies of the professions represented in this survey.

The respondents had collectively created apps with 33 functions within the last 5 years. By aggregating similar apps, 20 out of 33 (61%) sought to assist end users to self-manage or monitor chronic diseases, such as diabetes and mental health, while 8 (24%) apps intended to promote healthy lifestyles through diet and or exercise. The complete list of app functions can be found in Multimedia Appendix 2.

### Survey Responses According to mHAT Categories

In Table 1, we rank the *positive* (ie, sum of *completely agree* and *agree*), *neutral*, and *negative* (ie, sum of *completely disagree* and *disagree*) ratings according to categories of the checklist. Aside from one checklist item—“the app can inform end users about errors in measurements”—the combined positive ratings (ie, *completely agree* and *agree*) of the checklist items overwhelmingly exceeded the combined negative ratings (ie, *completely disagree* and *disagree*). The individual ratings for *completely disagree*, *disagree*, *neutral*, *agree*, and *completely agree* can be retrieved from Multimedia Appendix 3.

**Table 1:** Rankings of respondent ratings of checklist items.

Item			Negative, n (%)	Neutral, n (%)	Positive, n (%)
**Informational content (n=29)**					
	The privacy policies accompanying the app can be concise, clear, and easy to understand		1 (3)	6 (21)	22 (76)
	The terms of service accompanying the app can be concise and easy to read		1 (3)	7 (24)	21 (72)
	The app can be programmed such that it does not require too many end-user personal data		2 (7)	6 (21)	21 (72)
	The app can provide accurate measurements		3 (10)	5 (17)	21 (72)
	The app can be accompanied by clear end-user safety guidelines		3 (10)	6 (21)	20 (69)
	The app can be created with evidence from robust research		4 (14)	5 (17)	20 (69)
	The app can inform end users about errors in measurements		11 (38)	8 (28)	10 (34)
	**The app can recommend regular updates to:**				
		Fix bugs inherent within the app	5 (17)	4 (14)	20 (69)
		Amend app contents based on improved research	4 (14)	7 (24)	18 (62)
	**The information on the app can be certified by an:**				
		In-house team	4 (14)	6 (21)	19 (66)
		External third-party team	5 (17)	6 (21)	18 (62)
	The app can ensure that personalized data for end users is precise		1 (3)	12 (41)	16 (55)
	The research-backed evidence used to create the app can be easy to locate and understand		6 (21)	8 (28)	15 (52)
	The app can highlight potential risks or side effects resulting from its use		9 (31)	8 (28)	12 (41)
**Organizational attributes (n=28)**					
	My company can be transparent about our data-handling history and data breaches		2 (7)	4 (14)	22 (79)
	My company can demonstrate that it values data-protection regulations		2 (7)	4 (14)	22 (78)
	My company can employ skilled personnel within the app development domain to perform all tasks relating to the app		4 (14)	4 (14)	20 (71)
	My company can adopt clear policies on how to handle end-user data		3 (11)	6 (21)	19 (68)
	Our app can be affiliated with a nongovernmental organization or a reputable government agency		5 (18)	8 (29)	15 (54)
	My company has developed similar apps in the past		8 (29)	5 (18)	15 (54)
	My company has other reputable products or services to associate the app with		7 (25)	7 (25)	14 (50)
**Societal influences (n=25)**					
	End users can readily suggest the app to others		2 (8)	4 (16)	19 (76)
	The app store can display how often the app has been downloaded		3 (12)	5 (20)	17 (68)
	The app can display the positive reviews that it receives		5 (20)	6 (24)	14 (56)
	**To ensure that end users locate the app, it can be made to appear:**				
		In the top results of search engines	6 (24)	10 (40)	9 (36)
		As a featured app in the app store	4 (16)	10 (40)	11 (44)
	The app can accompany a wearable device		5 (20)	10 (40)	10 (40)
**Technology-related features (n=24)**					
	The app can be easy to use and have a friendly end-user interface		1 (4)	4 (17)	19 (79)
	The app can be made aesthetically appealing		1 (4)	5 (21)	18 (75)
	The app can be programmed to send out a reasonable number of notifications		1 (4)	5 (21)	18 (75)
	The data generated from the app can be anonymized to make individuals unidentifiable		3 (13)	3 (13)	18 (75)
	The app can be easily accessed by the end users it aims to target		1 (4)	5 (21)	18 (75)
	The data generated from the app can be secured by end-to-end encryption		0 (0)	7 (29)	17 (71)
	**The data generated from the app can be:**				
		Stored locally on the device	3 (13)	5 (21)	16 (67)
		Encrypted	1 (4)	7 (29)	16 (67)
	The app features can be customized by end users		4 (16)	5 (20)	15 (62)
	Privacy can be a core consideration throughout the life cycle of the app		4 (17)	7 (29)	13 (54)
	End users can easily access all of their data (eg, address and billing information)		4 (17)	7 (29)	13 (54)
**User control (n=23)**					
	The app can allow end users to easily delete their data		1 (4)	6 (26)	16 (70)
	The app can seek explicit end-user permission before sharing data with third parties		3 (13)	6 (26)	14 (61)
	The app can allow end users to opt in or decide which data can be stored or processed		2 (9)	7 (30)	14 (61)
	The app can give end users the freedom to control how their data are used		2 (9)	8 (35)	13 (57)
	The app can allow end users to restrict data sharing to third parties such as social networking sites		1 (4)	9 (39)	13 (57)
	The app can designate end users as proprietors (ie, owners) of their data		7 (30)	7 (30)	9 (39)

### Suggestions for Additional Survey Items

There were two new suggestions of items to include in the checklist: (1) transparency about business models and funding streams as well as (2) statistics on app uninstallations. These items were catalogued under the *organizational attributes (reputation)* and *informational content (transparency)* categories of the checklist, respectively. In the final version of the mHAT checklist found in Multimedia Appendix 4, the new items are indicated with an asterisk.

## Discussion

### Principal Findings

We have conducted a stakeholder survey to validate the mHAT checklist, a practical tool for app developers to create trustworthy mHealth apps. The checklist items can be considered feasible in many different settings, since the majority of the items were rated positively by the disparate stakeholders—UX researchers, administrators, IT personnel, health care professionals, researchers, managers, and marketing personnel—who reviewed its contents.

Throughout the survey, it appeared that those items that could apply to disparate mHealth apps were rated higher than those items that may be relevant to certain apps but not others. For example, “the app can inform end users about errors in measurements” was the most negatively rated item, whereas “the app can be easy to use and have a friendly end-user interface” was the most positively rated. One plausible explanation for this difference in ratings may be due to the aspiration to design any app, regardless of function, to be user friendly for its end users. The extent to which an app can have no measurement errors, however, will differ from the function of the app (eg, diabetes versus mental health).

The most negatively rated items in the survey—“the app can inform end users about error in measurements” and “the app can highlight potential risks or side effects resulting from its use”—demonstrated that app developers place marginal value in informing end users about the errors, risks, or side effects that may arise from their apps. Regardless of the reasons that contributed to these ratings, such information is vital to upholding end users’ trust in mHealth apps. Since plenty of evidence suggests that some apps on the market perpetuate inaccurate advice that may threaten patient safety, it is imperative that app developers are transparent about these issues [4,20]. Without a clear list of app risks or errors, end-user concerns about the trustworthiness of mHealth apps are bound to continue.

Since the checklist item “the app can inform end users about error in measurements” received more negative than positive ratings, it was excluded from the checklist. Meanwhile, respondents suggested including two new items: (1) a disclosure of the business model and (2) uninstallation statistics. Indeed, when app developers obscure their business models, they are perceived negatively by end users [6]. Including the uninstallation statistics of an app, however, is interesting for two reasons. On the one hand, it could signal transparency on the part of app developers and thus boost end users’ trust. On the other hand, it may deter end users from downloading the app altogether, since unpopular apps are unlikely to be downloaded in the first place [21].

### Strengths and Limitations

To the best of our knowledge, the mHAT checklist is the only empirically validated checklist that presents the attributes of trustworthy mHealth apps. This validated checklist is a robust tool for assisting app developers to create trustworthy apps. Our study has limitations. Despite multiple attempts to obtain a minimum sample size of 30 participants, challenges in recruiting participants meant that fewer participants could take part in the survey. Nonetheless, 23 participants answered all the survey items affording informative statistical analysis. The convenience sample recruited for this study is another limitation. Since these samples are usually unrepresentative of an entire population, our study may have missed out on capturing the opinions of some relevant stakeholders [22,23]. Further, ineligible individuals may have participated in the survey for two reasons: (1) it was an open survey and (2) to minimize access to respondents’ personal information, their computers were not assigned unique identification numbers. Nonetheless, there was no incentive to participate, since no compensation was awarded.

### Implications for Future Research

The mHAT checklist informs the conversation on the expectations of trustworthy mHealth apps. Future studies should assess whether the checklist is suitable for all mHealth apps or whether it is better suited for certain apps than others. Through additional studies, the contents of the checklist can be improved to retain useful items and exclude redundant ones. More enquiries about the underlying reasons why app developers see little value in informing end users about the risks, side effects, or errors of their apps is also warranted.

### Conclusions

This study presents a validated mHAT checklist: a useful guide for app developers to create trustworthy health apps. The 41-item checklist is comprised of five main categories—informational content, organizational attributes, societal influences, technology-related factors, and user control—and 11 subcategories—information accuracy, understandability, transparency, brand familiarity, reputation, recommendations, external factor, usability, privacy, autonomy, and empowerment (see Multimedia Appendix 4).

## Appendix

Multimedia Appendix 1 The unvalidated mobile health app trustworthiness (mHAT) checklist.

Multimedia Appendix 2 Functions of apps developed by respondents.

Multimedia Appendix 3 Frequencies and percent ratings of survey items.

Multimedia Appendix 4 The updated mobile health app trustworthiness (mHAT) checklist. New items to the checklist are indicated with an asterisk.

## Abbreviations

ETH Zurich: Swiss Federal Institute of Technology
IP: Internet Protocol
IT: information technology
mHAT: mobile health app trustworthiness
mHealth: mobile health
UX: user experience
