# Supplementary material for: The Mobile Health App Trustworthiness Checklist: Usability Assessment
Source: JMIR Mhealth Uhealth. 2020 Jul 21;8(7):e16844. doi: 10.2196/16844 (PMC7404005; doi:10.2196/16844)
Supplement: Multimedia Appendix 1 [file mhealth_v8i7e16844_app1.pdf]

## Appendix 1: The unvalidated mHealth App Trustworthiness (mHAT) Checklist

| | Question | Yes | No | Not applicable | In progress | Comments |
|---|---|---|---|---|---|---|
| **Informational Content** | | | | | | |
| **Information accuracy** | Does the app provide accurate measurements? | ☐ | ☐ | ☐ | ☐ | |
| | Does the app inform end-users about errors in measurements? | ☐ | ☐ | ☐ | ☐ | |
| | Does the app ensure that personalized data tailored to end-users are precise? | ☐ | ☐ | ☐ | ☐ | |
| | Is the information on the app certified by an:<br>a. in-house team?<br>b. external third-party team? | ☐<br>☐ | ☐<br>☐ | ☐<br>☐ | ☐<br>☐ | |
| | Is the information provided by the app backed by robust research? | ☐ | ☐ | ☐ | ☐ | |
| | Does the app recommend regular updates to:<br>a. fix bugs inherent within the app?<br>b. amend app contents based on improved research? | ☐<br>☐ | ☐<br>☐ | ☐<br>☐ | ☐<br>☐ | |
| **Understandability** | Is the app accompanied by clear end-user safety guidelines? | ☐ | ☐ | ☐ | ☐ | |
| | Is the research-backed evidence used to create the app easy to locate and understand? | ☐ | ☐ | ☐ | ☐ | |
| **Transparency** | Does the app highlight potential risks or side-effects resulting from its use? | ☐ | ☐ | ☐ | ☐ | |
| | Are the ‘terms of service’ concise and easy to read? | ☐ | ☐ | ☐ | ☐ | |
| | Does the app require only minimal personal data of end-users?* | ☐ | ☐ | ☐ | ☐ | |
| | Are the privacy policies concise, clear and easy to understand? | ☐ | ☐ | ☐ | ☐ | |
| **Organizational Attributes** | | | | | | |
| **Brand familiarity** | Does the company have other reputable products or services to associate the app with? | ☐ | ☐ | ☐ | ☐ | |
| **Reputation** | Does the company curating the app have clear policies on how to handle end-user data? | ☐ | ☐ | ☐ | ☐ | |
| | Does the company make their data handling history and data breaches available to end-users? | ☐ | ☐ | ☐ | ☐ | |
| | Is the app affiliated with a non-governmental organization or a reputable government agency?* | ☐ | ☐ | ☐ | ☐ | |
| | Does the company value data protection regulations? | ☐ | ☐ | ☐ | ☐ | |
| | Does the company utilize skilled personnel within the app development domain? | ☐ | ☐ | ☐ | ☐ | |

| | | | | | | |
|---|---|---|---|---|---|---|
| | Has the company developed similar apps in the past? | ☐ | ☐ | ☐ | ☐ | |
| **Societal Influences** | | | | | | |
| Recommendations | Can end-users readily suggest the app to others? | ☐ | ☐ | ☐ | ☐ | |
| | Does the app have good reviews? | ☐ | ☐ | ☐ | ☐ | |
| | How easily can end-users locate the app? Does it appear:<br>a. in the top results of search engines?<br>b. as a featured app in the app store? | ☐<br>☐ | ☐<br>☐ | ☐<br>☐ | ☐<br>☐ | |
| | Does the app store display how often the app has been downloaded? | ☐ | ☐ | ☐ | ☐ | |
| External Factor | Does the app accompany a wearable device? | ☐ | ☐ | ☐ | ☐ | |
| **Technology-related Features** | | | | | | |
| Usability | Is the app easy to use and have a friendly end-user interface? | ☐ | ☐ | ☐ | ☐ | |
| | Is the app visually appealing (aesthetics)? | ☐ | ☐ | ☐ | ☐ | |
| | Does the app send out a reasonable number of notifications?* | ☐ | ☐ | ☐ | ☐ | |
| | Are the features of the app customizable? | ☐ | ☐ | ☐ | ☐ | |
| | Is the app accessible by its target audience?* | ☐ | ☐ | ☐ | ☐ | |
| Privacy | Is the data generated from the app secured by end-to-end-encryption? | ☐ | ☐ | ☐ | ☐ | |
| | How is the data generated from the app stored?<br>a. locally on the device?<br>b. encrypted? | ☐<br>☐ | ☐<br>☐ | ☐<br>☐ | ☐<br>☐ | |
| | Is privacy a core consideration throughout the app design phase? i.e. a privacy by design approach | ☐ | ☐ | ☐ | ☐ | |
| | Is the data generated from the app anonymized so individuals are non-identifiable? | ☐ | ☐ | ☐ | ☐ | |
| | Can users easily access all of their data e.g. address, billing information? | ☐ | ☐ | ☐ | ☐ | |
| **User Control** | | | | | | |
| Autonomy | Do the functions of the app give end-users the overall impression of freedom to control the use of their data? | ☐ | ☐ | ☐ | ☐ | |
| Empower-ment | Does the app allow end-users to restrict data sharing to third-parties such as social networking sites? | ☐ | ☐ | ☐ | ☐ | |
| | Do end-users act as the proprietors of the data generated from the app? | ☐ | ☐ | ☐ | ☐ | |

| | | | | | | |
|---|---|---|---|---|---|---|
| | Does the app seek explicit end-user permission before sharing data with third-parties? | ☐ | ☐ | ☐ | ☐ | |
| | Does the app allow end-users to opt-in and decide which data can be stored or processed?* | ☐ | ☐ | ☐ | ☐ | |
| | Does the app allow end-users to easily delete their data? | ☐ | ☐ | ☐ | ☐ | |

*Note: the relevance of the checklist items marked by asterisks (*) may vary depending on app function*
