# Supplementary material for: The Mobile Health App Trustworthiness Checklist: Usability Assessment
Source: JMIR Mhealth Uhealth. 2020 Jul 21;8(7):e16844. doi: 10.2196/16844 (PMC7404005; doi:10.2196/16844)
Supplement: Multimedia Appendix 2 [file mhealth_v8i7e16844_app2.docx]

**Appendix 2:** **functions of apps developed by respondents**

| - Test - Post-surgery monitoring of patients that had bariatric surgery^1^ - Prevention of secondary health conditions and improvement of self-management^1^ - Symptom monitoring^1^ - Reducing salt intake^1^ - Helping junior doctors with daily activities on the ward such as ordering scans - Promoting exercise^2^ - Arthritis self-monitoring^1^ - Supporting cancer patients with adverse events^1^ - Promoting self-management of cancer-related fatigue^1^ - Risk assessment^1^ - Cancer prevention^1^ - Diabetes^1^ - Diabetes^1^ - Type 2 diabetes management Type 2 diabetes prevention Weight management^1^ - Mental health^1^ - Migraine and headache management^1^ - Track food and drink consumption^2^ - Evaluation^1^ - Mental health risk assessment and management^1^ - Allowing citizens access to services - Exercise routine - Promoting exercise^2^ - Programming - Promoting exercise^2^ - Tracking amount of daily steps and miles per day^2^ - Exercise ^2^ - Fitbit ^2^ - Exercise ^2^ - Diabetes management^1^ - Promoting healthier lifestyle^2^ - Management of diseases^1^ - Marketing the app to the public - Blood donation and management^1^ - Update patients on continuity of care^1^ |
| --- |

*Note: ^1^ apps to self-manage or monitor conditions; ^2^ apps to promote healthy lifestyles*
