# Supplementary material for: The Mobile Health App Trustworthiness Checklist: Usability Assessment
Source: JMIR Mhealth Uhealth. 2020 Jul 21;8(7):e16844. doi: 10.2196/16844 (PMC7404005; doi:10.2196/16844)
Supplement: Multimedia Appendix 3 [file mhealth_v8i7e16844_app3.docx]

**Appendix 3: Frequencies and percent ratings of survey items**

| **Question** | | -- | - | 0 | + | ++ |
| --- | --- | --- | --- | --- | --- | --- |
| **Informational Content** (n = 29) | | | | | | |
|  | The app can provide accurate measurements | 1 | 2 | 5 | 14 | 7 |
|  |  | 3.4% | 6.9% | 17.2% | 48.3% | 24.1% |
|  | The app can inform end-users about errors in measurements | 3 | 8 | 8 | 8 | 2 |
|  |  | 10.3% | 27.6% | 27.6% | 27.6% | 6.9% |
|  | The app can ensure that personalized data tailored to end-users is precise | 1 | 0 | 12 | 9 | 7 |
|  |  | 3.4% | 0.0% | 41.4% | 31.0% | 24.1% |
|  | The information on the app can be certified by an: | 1 | 3 | 6 | 14 | 5 |
|  | In-house team? | 3.4% | 10.3% | 20.7% | 48.3% | 17.2% |
|  |  | 2 | 3 | 6 | 10 | 8 |
|  | External third-party team? | 6.7% | 10.3% | 20.7% | 34.5% | 27.6% |
|  | The app can be created with evidence from robust research | 2 | 2 | 5 | 10 | 10 |
|  |  | 6.9% | 6.9% | 17.2% | 34.5% | 34.5% |
|  | The app can recommend regular updates to: | 2 | 3 | 4 | 12 | 8 |
|  | Fix bugs found within the app | 6.9% | 10.3% | 13.8% | 41.3% | 27.6% |
|  |  | 2 | 2 | 7 | 10 | 8 |
|  | Amend its contents based on updated research | 6.9% | 6.9% | 24.1% | 34.5% | 27.6% |
|  | The app can be accompanied by clear end-user safety guidelines | 1 | 2 | 6 | 11 | 9 |
|  |  | 3.4% | 6.7% | 20.7% | 37.9% | 31.0% |
|  | The research-backed evidence used to create the app can be easy to locate and understand | 1 | 5 | 8 | 7 | 8 |
|  |  | 3.4% | 17.2% | 27.6% | 24.1% | 27.6% |
|  | The app can highlight the potential risks or side-effects resulting from its use | 4 | 5 | 8 | 9 | 3 |
|  |  | 13.8% | 17.2% | 27.6% | 31.0% | 10.3% |
|  | The terms of service accompanying the app can be concise and easy to read | 0 | 1 | 7 | 11 | 10 |
|  |  | 0.0% | 3.4% | 24.1% | 37.9% | 34.5% |
|  | The app can be programmed such that it does not require too many end-user personal details | 0 | 2 | 6 | 10 | 11 |
|  |  | 0.0% | 6.9% | 20.7% | 34.5% | 37.9% |
|  | The privacy policies accompanying the app can be concise, clear and easy to understand | 0 | 1 | 6 | 11 | 11 |
|  |  | 0.0% | 3.4% | 20.7% | 37.9% | 37.9% |
| **Organizational Attributes** (n = 28) | | | | | | |
|  | My company has other reputable products or services to associate the app with | 2 | 5 | 7 | 6 | 8 |
|  |  | 7.1% | 17.9% | 25.0% | 21.4% | 28.6% |
|  | My company can adopt clear policies on how to handle end-user data | 1 | 2 | 6 | 8 | 11 |
|  |  | 3.4% | 7.1% | 21.4% | 28.6% | 39.2% |
|  | My company can be transparent about our data handling history and data breaches | 1 | 1 | 4 | 10 | 12 |
|  |  | 3.4% | 3.4% | 14.3% | 35.7% | 42.9% |
|  | Our app can be affiliated with a non-governmental organization or a reputable government agency | 2 | 3 | 8 | 7 | 8 |
|  |  | 7.1% | 10.7% | 28.6% | 25.0% | 28.6% |
|  | My company can demonstrate that it values data protection regulations | 1 | 1 | 4 | 12 | 10 |
|  |  | 3.4% | 3.4% | 14.3% | 42.9% | 35.7% |
|  | My company can employ skilled personnel within the app development domain to perform all tasks relating to the app | 1 | 3 | 4 | 10 | 10 |
|  |  | 3.6% | 10.7% | 14.3% | 35.7% | 35.7% |
|  | My company has developed similar apps in the past | 2 | 6 | 5 | 8 | 7 |
|  |  | 7.1% | 21.4% | 17.9% | 28.6% | 25.0% |
| **Societal Influence** (n = 25) | | | | | | |
|  | End-users can readily suggest the app to others | 1 | 1 | 4 | 14 | 5 |
|  |  | 4.0% | 4.0% | 16.0% | 56.0% | 20.0% |
|  | The app can display the positive reviews that it receives | 2 | 3 | 6 | 7 | 7 |
|  |  | 8.0% | 12.0% | 24.0% | 28.0% | 28.0% |
|  | To ensure that end-users can easily locate the app, it can be made to appear: | 3 | 3 | 10 | 6 | 3 |
|  | In the top results of search engines |  |  |  |  |  |
|  |  | 12.0% | 12.0% | 40.0% | 24.0% | 12.0% |
|  | As a featured app in the app store | 3 | 1 | 10 | 6 | 5 |
|  |  | 12.0% | 4.0% | 40.0% | 24.0% | 20.0% |
|  | The app store can display how often the app has been downloaded | 2 | 1 | 5 | 9 | 8 |
|  |  | 8.0% | 4.0% | 20.0% | 36.0% | 32.0% |
|  | The app can accompany a wearable device | 5 | 0 | 10 | 6 | 4 |
|  |  | 20.0% | 0.0% | 40.0% | 24.0% | 16.0% |
| **Technological Influence** (n = 24) | | | | | | |
|  | The app can be easy to use and have a friendly end-user interface | 0 | 1 | 4 | 7 | 12 |
|  |  | 0.0% | 4.2% | 16.7% | 29.2% | 50.0% |
|  | The app can be made aesthetically appealing | 0 | 1 | 5 | 7 | 11 |
|  |  | 0.0% | 4.2% | 20.8% | 29.2% | 45.8% |
|  | The app can be programmed to send out only a reasonable number of notifications | 0 | 1 | 5 | 7 | 11 |
|  |  | 0.0% | 4.2% | 20.8% | 29.2% | 45.8% |
|  | The app features can be customized by end-users | 1 | 3 | 5 | 9 | 6 |
|  |  | 4.2% | 12.5% | 20.8% | 37.5% | 25.0% |
|  | The app can be easily accessed by the end-users it aims to target | 0 | 1 | 5 | 11 | 7 |
|  |  | 0.0% | 4.2% | 20.8% | 45.8% | 29.2% |
|  | The data generated from the app can be secured by end-to-end encryption | 0 | 0 | 7 | 6 | 11 |
|  |  | 0.0% | 0.0% | 29.2% | 25.0% | 45.8% |
|  | The data generated from the app can be stored: | 1 | 2 | 5 | 9 | 7 |
|  | Locally on the device | 4.2% | 8.3% | 20.8% | 37.5% | 29.2% |
|  | Encrypted | 0 | 1 | 7 | 8 | 8 |
|  |  | 0.0% | 4.2% | 29.2% | 33.3% | 33.3% |
|  | Privacy can be a core consideration throughout the life cycle of the app | 0 | 4 | 7 | 4 | 9 |
|  |  | 0.0% | 16.7% | 29.2% | 16.7% | 37.5% |
|  | The data generated from the app can be anonymized to make individuals unidentifiable | 0 | 3 | 3 | 9 | 9 |
|  |  | 0.0% | 12.5% | 12.5% | 37.5% | 37.5% |
|  | End-users can easily access all of their data e.g. address & billing information | 2 | 2 | 7 | 7 | 6 |
|  |  | 8.3% | 8.3% | 29.2% | 29.2% | 25.0% |
| **User Control** (n = 23) | | | | | | |
|  | The app can give end-users the freedom to control how their data is used | 0 | 2 | 8 | 7 | 6 |
|  |  | 0.0% | 8.7% | 34.8% | 30.4% | 26.1% |
|  | The app can allow end-users to restrict data sharing to third-parties such as social networking sites | 0 | 1 | 9 | 4 | 9 |
|  |  | 0.0% | 4.4% | 39.1% | 17.4% | 39.1% |
|  | The app can designate end-users as the proprietors (owners) of their data | 2 | 5 | 7 | 6 | 3 |
|  |  | 8.7% | 21.7% | 30.4% | 26.1% | 13.0% |
|  | The app can seek explicit end-user permission before sharing any data with third-parties | 1 | 2 | 6 | 7 | 7 |
|  |  | 4.4% | 8.7% | 26.1% | 30.4% | 30.4% |
|  | The app can allow end-users to opt-in or decide which data can be stored or processed | 0 | 2 | 7 | 11 | 3 |
|  |  | 0.0% | 8.7% | 30.4% | 47.8% | 13.0% |
|  | The app can allow end-users to easily delete their data | 0 | 1 | 6 | 9 | 7 |
|  |  | 0.0% | 4.4% | 26.1% | 39.1% | 30.4% |

*Note: Completely disagree (--), disagree (-), neutral (0), agree (+) and completely agree (++)*
