# Supplementary material for: The Mobile Health App Trustworthiness Checklist: Usability Assessment
Source: JMIR Mhealth Uhealth. 2020 Jul 21;8(7):e16844. doi: 10.2196/16844 (PMC7404005; doi:10.2196/16844)
Supplement: Multimedia Appendix 4 [file mhealth_v8i7e16844_app4.docx]

| **Question** | | **Yes** | **No** | **Not applicable** | **In progress** | **Comments** |
| --- | --- | --- | --- | --- | --- | --- |
|  | | | | | | |
| **Informational Content** | | | | | | |
| **Information Accuracy** | Does the app provide accurate measurements? |  |  |  |  |  |
|  | Does the app ensure that personalized data tailored to end users are precise? |  |  |  |  |  |
|  | Is the information on the app certified by an:   - In-house team? - External third-party team? |  |  |  |  |  |
|  | Is the information provided by the app backed by robust research? |  |  |  |  |  |
|  | Does the app recommend regular updates to:   - Fix bugs inherent within the app? - Amend app contents based on improved research? |  |  |  |  |  |
| **Understandability** | Is the app accompanied by clear end-user safety guidelines? |  |  |  |  |  |
|  | Is the research-backed evidence used to create the app easy to locate and understand? |  |  |  |  |  |
| **Transparency** | Does the app highlight potential risks or side effects resulting from its use? |  |  |  |  |  |
|  | Are the terms of service concise and easy to read? |  |  |  |  |  |
|  | Does the app require only minimal personal data of end users? |  |  |  |  |  |
|  | Are the privacy policies concise, clear, and easy to understand? |  |  |  |  |  |
|  | Does the app provide details about uninstallation statistics? * |  |  |  |  |  |
| **Organizational Attributes** | | | | | | |
| **Brand Familiarity** | Does the company have other reputable products or services to associate the app with? |  |  |  |  |  |
| **Reputation** | Does the company curating the app have clear policies on how to handle end-user data? |  |  |  |  |  |
|  | Does the company make their data-handling history and data breaches available to end users? |  |  |  |  |  |
|  | Is the app affiliated with a nongovernmental organization or a reputable government agency? |  |  |  |  |  |
|  | Does the company value data-protection regulations? |  |  |  |  |  |
|  | Does the company utilize skilled personnel within the app development domain? |  |  |  |  |  |
|  | Has the company developed similar apps in the past? |  |  |  |  |  |
|  | Does the company provide details about the business and funding model of the app? * |  |  |  |  |  |
| **Societal Influences** | | | | | | |
| **Recommendations** | Can end users readily suggest the app to others? |  |  |  |  |  |
|  | Does the app have good reviews? |  |  |  |  |  |
|  | How easily can end users locate the app? Does it appear:   - In the top results of search engines? - As a featured app in the app store? |  |  |  |  |  |
|  | Does the app store display how often the app has been downloaded? |  |  |  |  |  |
| **External Factor** | Does the app accompany a wearable device? |  |  |  |  |  |
| **Technology-Related Features** | | | | | | |
| **Usability** | Is the app easy to use and does it have a friendly end-user interface? |  |  |  |  |  |
|  | Is the app visually appealing (ie, aesthetics)? |  |  |  |  |  |
|  | Does the app send out a reasonable number of notifications? |  |  |  |  |  |
|  | Are the features of the app customizable? |  |  |  |  |  |
|  | Is the app accessible by its target audience? |  |  |  |  |  |
| **Privacy** | Are the data generated from the app encrypted? |  |  |  |  |  |
|  | How is the data generated from the app stored:   - Locally on the device? - Encrypted? |  |  |  |  |  |
|  | Is privacy a core consideration throughout the app design phase (ie, a privacy-by-design approach)? |  |  |  |  |  |
|  | Are the data generated from the app anonymized so individuals are nonidentifiable? |  |  |  |  |  |
|  | Can users easily access all their data (eg, address and billing information)? |  |  |  |  |  |
| **User Control** | | | | | | |
| **Autonomy** | Do the functions of the app give end users the overall impression of freedom to control the use of their data? |  |  |  |  |  |
| **Empowerment** | Does the app allow end users to restrict data sharing to third parties such as social networking sites? |  |  |  |  |  |
|  | Do end users act as the proprietors of the data generated from the app? |  |  |  |  |  |
|  | Does the app seek explicit end-user permission before sharing data with third parties? |  |  |  |  |  |
|  | Does the app allow end users to opt in and decide which data can be stored or processed? |  |  |  |  |  |
|  | Does the app allow end users to easily delete their data? |  |  |  |  |  |
